# Up-regulation of Toll-like receptors 7 and 9 and its potential implications in the pathogenic mechanisms of *LMNA*-related myopathies

**DOI:** 10.1080/19491034.2018.1471947

**Published:** 2018-10-03

**Authors:** Cristina Cappelletti, Franco Salerno, Eleonora Canioni, Marina Mora, Renato Mantegazza, Pia Bernasconi, Lorenzo Maggi

**Affiliations:** Neurology IV - Neuroimmunology and Neuromuscular Diseases Unit, Fondazione IRCCS Istituto Neurologico “Carlo Besta”, Milan, Italy

**Keywords:** Laminopathies, Toll-like receptors, macrophages, skeletal muscle, muscle damage

## Abstract

Laminopathies are a heterogeneous group of diseases, caused by mutations in lamin A/C proteins. The most common laminopathy (*LMNA*-related myopathies, *LMNA-RM*) affects skeletal and cardiac muscles; muscle histopathology is variable, ranging from mild unspecific changes to dystrophic features, sometimes with inflammatory evidence. Whether the genetic defect might activate innate immune components, leading to chronic inflammation, myofiber necrosis and fibrosis, is still unknown. By qPCR, a significant up-regulation of Toll-like receptor (TLR) 7 and 9 transcripts was found in *LMNA*-RM compared to other myopathic and non-myopathic muscles. A marked TLR7/9 staining was observed on *LMNA*-RM blood vessels and muscle fibers and, when present, on infiltrating cells, mainly macrophages, scattered in the tissue or localized close to degenerated muscle fibers and connective tissue. Our results recognize innate immunity as a player in *LMNA*-RM pathogenesis. Modulation of TLR7/9 signaling pathways and decrease of macrophage-mediated inflammation might be potential therapeutic strategies in *LMNA*-RM management.

**Abbreviations**: DMD, Duchenne muscular dystrophy; EDMD2, Emery-Dreifuss muscular dystrophy type 2; FSHD, facio-scapulo-humeral muscular dystrophy; LGMD1B, limb-girdle muscular dystrophy type 1B; *LMNA*-CMD, *LMNA-*related congenital muscular dystrophy; *LMNA*-RM, *LMNA*-related myopathies; sIBM, sporadic inclusion body myositis; TLR, Toll-like receptor

## Introduction

The lamin A and C proteins, derived from alternative splicing of the *LMNA* gene, are intermediate filament proteins that are components of the internal nuclear lamina [1]. Based on the affected tissue, laminopathies can be classified into several categories: lipodystrophies, neuropathies, dermopathies, cardiomyopathies and muscular dystrophies [2]. Muscular phenotypes associated with *LMNA* mutations (*LMNA*-RM) include three main groups based on the distribution of muscle weakness or age at onset: limb-girdle muscular dystrophy type 1B (LGMD1B), Emery-Dreifuss muscular dystrophy type 2 (EDMD2) and a form of congenital muscular dystrophy (*LMNA*-CMD) [3]. In *LMNA*-RM affected subjects, histological findings in the muscle tissue are very variable ranging from mild and unspecific changes to dystrophic features, sometimes with inflammatory infiltrates [4–6]. Of note, inflammatory cells, which may be scattered or localized close to degenerated myofibers and to connective tissue deposits, seem to be more frequent in muscle samples taken from *LMNA*-RM patients with onset in the first years of life than in muscles from *LMNA*-RM patients with adult onset [4,7].

As recently demonstrated in Duchenne muscular dystrophy (DMD) [8], genetic defect may affect muscle fiber structure and promote the release of adjuvant stimuli triggering the activation of innate and adaptive immune responses. Regarding laminopathies, it was demonstrated that myoblasts from *LMNA* mutated patients secrete high levels of inflammatory cytokines, such as IL-6 and IL-8 [9], resembling senescent cells [10] or persistently damaged cells [11]. This ongoing inflammatory state might recruit and activate immune cells as well as Toll-like receptors (TLRs) contributing to muscle degeneration, and strongly influencing the development of muscle fibrosis [12,13]. TLRs, particularly TLR4, TLR7 and TLR9, are mainly expressed by macrophages, and other lymphocytes, e.g. myeloid dendritic cells; once activated, they trigger a cascade of signaling pathways leading to the induction of several pro- or anti-inflammatory cytokine genes. In genetic muscular disorders, especially in DMD, the increased and persistent presence of macrophages, in addition to the over-expression of TLR7, it has been proposed to highly contribute to myofiber necrosis and fibrosis [8,12]. The switch from pro- to anti-inflammatory macrophage phenotype and the continuous release of TGF-β, promote fibroblast proliferation and connective tissue deposition, in addition to the increase availability of proline for collagen synthesis [14].

Recent reports provided evidence for a critical role of lamins in muscle differentiation [2,15]. Indeed, mutated lamin A/C or emerin negatively affect the expression of genes necessary for muscle cell differentiation, such as MyoD, desmin, pRb and M-cadherin [16].

In view of these considerations, in the present work we proposed to evaluate the expression and localization of endosomal TLR7 and TLR9 in a group of *LMNA*-RM affected patients, featuring both dystrophic and myopathic phenotype and distinct age of onset. We further characterized the discernible inflammatory cells in order to assess the percentage of TLR7/9 positive immune cells. Finally, we evaluated the expression of TGF-β and its potential relationship with damaged muscle fibers. *LMNA*-RM data were compared to those obtained in sporadic inclusion body myositis (sIBM) and facio-scapulo-humeral muscular dystrophy (FSHD), considered as pathological controls. sIBM is an inflammatory myopathy, characterized by a massive muscle infiltration of mononuclear cells and an activation of innate immunity TLR-mediated [17,18]; FSHD represents a genetically heterogeneous form of muscle dystrophy, characterized histologically by a myopathic or dystrophic muscle with mononuclear cell infiltrates [19,20]. Furthermore, it has been demonstrated that *DUX4* expression in muscle cells is involved in the regulation of immune mediators, such as TLR4 [21].

## Results

### Increase of TLR7 and TLR9 mRNA expression levels in *LMNA*-RM and sIBM compared to FSHD and control muscles

**Figure 1.**
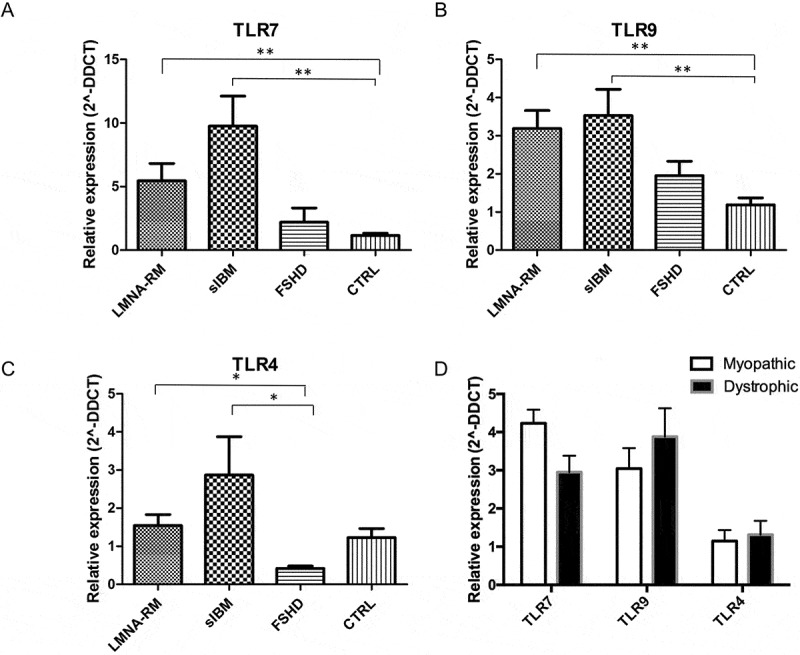
Expression of TLR7, TLR9 and TLR4 in *LMNA*-RM, sIBM, FSHD patients and non-myopathic controls (CTRL) was quantified by qRT-PCR. (a) and (b) show that TLR7 and TLR9 were significantly up-regulated in *LMNA*-RM and sIBM, but not in FSHD, compared to controls. (c) TLR4 expression levels were comparable between patients and controls; a statistically significant difference was observed between *LMNA*-RM and FSHD and between sIBM and FSHD. (d) Comparison between dystrophic and myopathic *LMNA*-RM patients did not show any significant difference in the mRNA expression levels of the TLRs analysed. Data are represented as mean ± SEM. *p < 0.05, **p < 0.01.

At first we evaluated the mRNA expression levels of TLR7, TLR9 and TLR4 in muscle tissue of *LMNA*-RM patients and the results were compared with those obtained from sIBM, FSHD and non-myopathic control muscles (Figure 1). We found that TLR7 and TLR9 relative expression values (± SEM) were significantly higher in *LMNA*-RM and sIBM than in control muscles (5.46 ± 1.36 and 9.75 ± 2.37, respectively, versus 1.15 ± 0.18 for TLR7, *p* < 0.01; 3.19 ± 0.47 and 3.53 ± 0.69 versus 1.19 ± 0.18 for TLR9, *p* < 0.01), whereas in FSHD the expression levels of both receptors were comparable with those of non-myopathic controls (Figure 1(a,b)) (2.19 ± 1.13 for TLR7 and 1.95 ± 0.38 for TLR9). TLR4 mRNA expression levels significantly differed in *LMNA*-RM and sIBM compared to FSHD, but not to controls (Figure 1(c)) (*LMNA*-RM: 1.55 ± 0.29; sIBM: 2.87 ± 1.01; FSHD: 0.42 ± 0.06; controls: 1.23 ± 0.23, *p* < 0.05). Within *LMNA*-RM group, no significant difference in TLR7 and 9 expression was observed between dystrophic and myopathic patients (Figure 1(d)) (2.95 ± 0.43 versus 4.23 ± 0.36 for TLR7, *p* = 0.23; 3.88 ± 0.74 versus 3.04 ± 0.54 for TLR9, *p* = 0.40). Therefore, these data suggest that the TLR pathway is not associated with the severity of histological findings.

### Immunohistochemistry of TLR7, TLR9 and TLR4 in *LMNA*-RM muscle tissue

**Figure 2.**
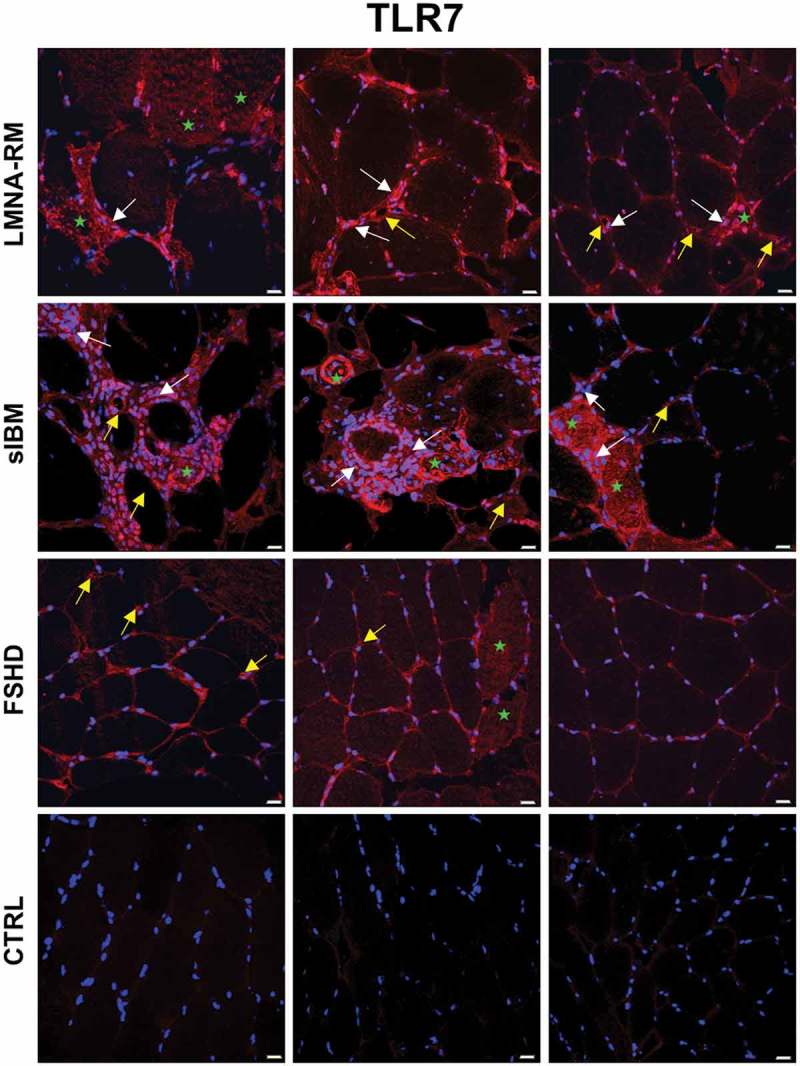
Immunofluorescence staining of TLR7 in *LMNA*-RM, sIBM, FSHD patient and non-myopathic control (CTRL) muscles. In *LMNA*-RM TLR7 was highly expressed in the endomysial space, on capillaries and small blood vessels (yellow arrows), at the level of discernable inflammatory cells (white arrows), and occasionally on the sarcolemma or within some muscle fibers (green stars). In sIBM TLR7 localized mainly at the level of inflammatory infiltrates (white arrows) and on degenerating myofibers (green stars) surrounded/invaded by immune infiltrates or characterized by sarcoplasmic inclusions. FSHD showed a TLR7 positive staining in the endomysium and rarely within some muscle fibers (green stars). A weak or negative staining was observed in control muscle tissues. Original magnification, X40; bar 20 μm.

**Figure 3.**
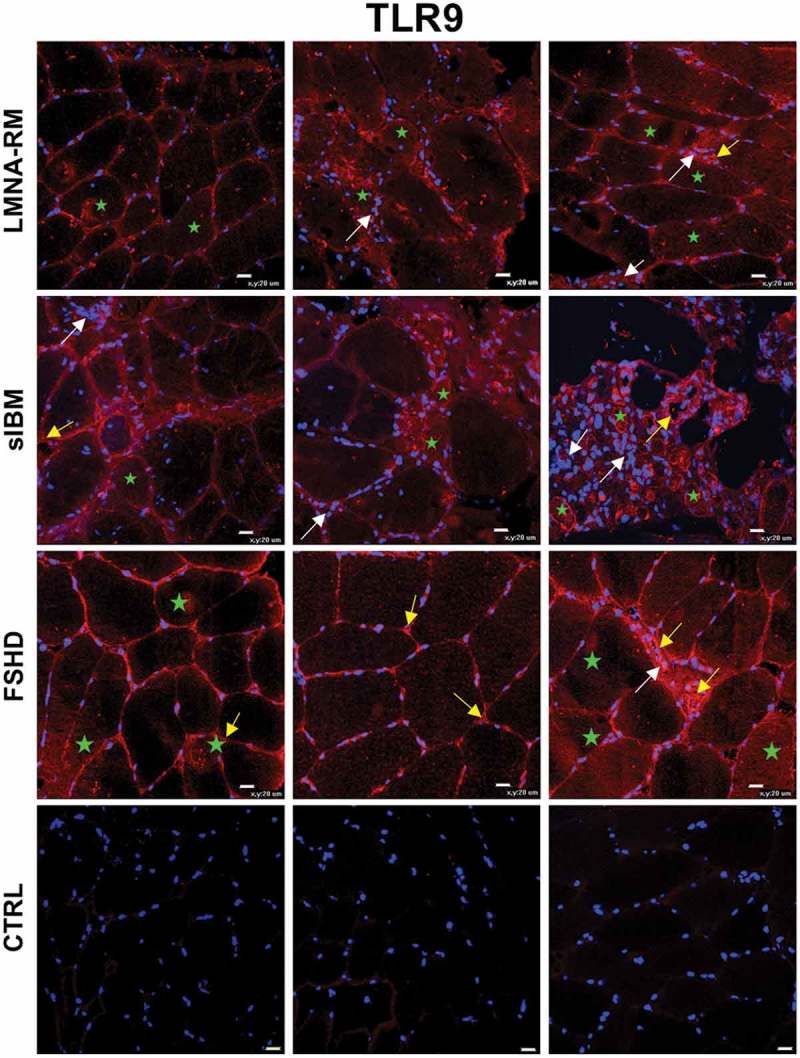
TLR9 expression and localization in *LMNA*-RM, sIBM, FSHD patients and non-myopathic control (CTRL) muscle tissues. All myopathic samples showed a positive immunoreactivity for TLR9. The receptor was mainly detected in the endomysial space, on the sarcolemma and sometimes within myofibers (green stars), on inflammatory cells (white arrows) and on capillaries and small blood vessels (yellow arrows). A weak or negative staining was observed in control muscle tissues. Original magnification, X40; bar 20 μm.

**Figure 4.**
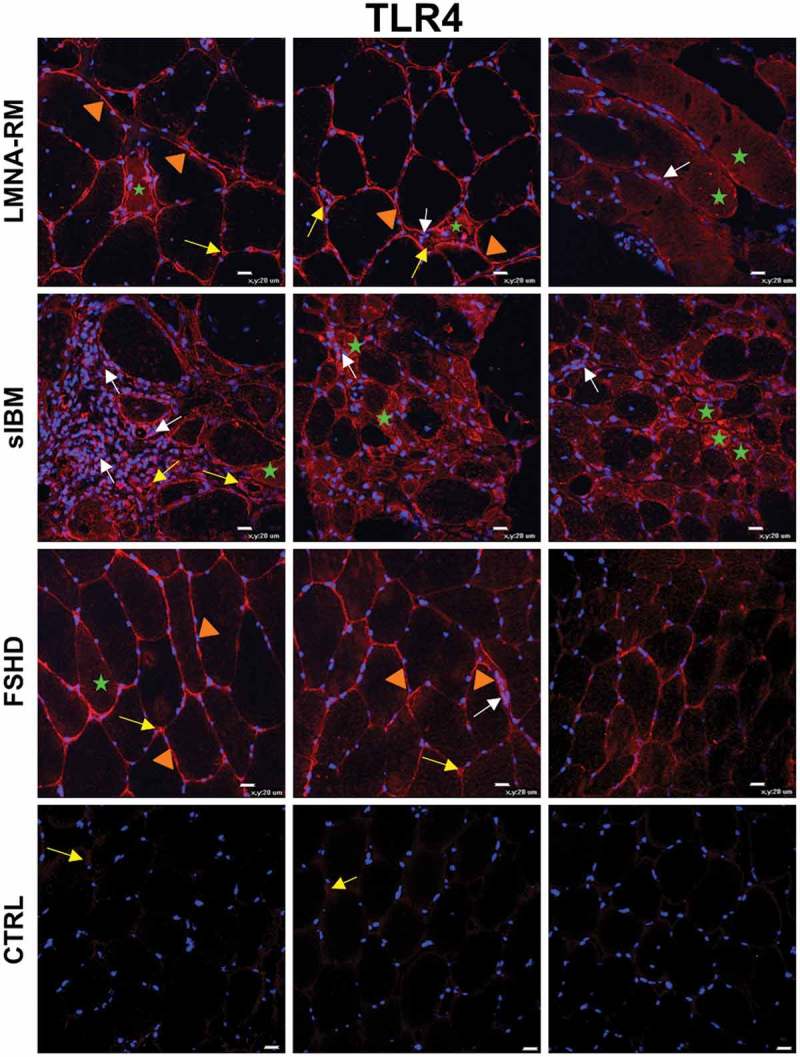
Immunofluorescence of TLR4 in *LMNA*-RM, sIBM, FSHD patients and non-myopathic control (CTRL) muscles. In *LMNA*-RM, sarcolemma (orange arrowheads), sporadically sarcoplasm (green stars) of some muscle fibers and capillaries (yellow arrows) were highly positive for TLR4. In sIBM, TLR4 was strongly expressed within small sized degenerated/regenerated myofibers (green stars), at the level of muscle infiltrating cells (white arrows) and on capillaries (yellow arrows). FSHD showed a TLR4 positive staining mainly on the sarcolemma of some muscle fibers (orange arrowheads), rarely in the sarcoplasm (green star) and on capillaries (yellow arrows), whereas in control muscle tissues a weak TLR4 immunopositivity was observed on capillaries (yellow arrows). Original magnification, X40; bar 20 μm.

The expression and localization of endosomal TLRs were evaluated in 4 *LMNA*-RM, 4 sIBM, 3 FSHD and 3 non-myopathic control muscle tissues by fluorescence microscopy (Figures 2–4). In *LMNA*-RM muscle tissue TLR7 was mostly expressed in the endomysial space, on capillaries and small blood vessels, and at the level of some inflammatory cells scattered in the sample or localized close to damaged muscle fibers and connective tissue deposits (Figure 2). Occasionally, a positive staining for TLR7 was observed on the sarcolemma or within some degenerated muscle fibers, mainly in dystrophic *LMNA*-RM muscle tissues (Supplementary Figure 1). In sIBM, we found that TLR7 mainly localized at the level of inflammatory infiltrates, on the sarcolemma and within degenerating myofibers characterized by inclusion bodies or surrounded by muscle infiltrating cells, and on capillaries and small blood vessels (Figure 2). In FSHD, TLR7 was mainly expressed in the endomysium among muscle fibers, on capillaries and small blood vessels, and rarely in the sarcoplasm of myofibers (Figure 2).

Unlike TLR7, in all myopathic muscles TLR9 was expressed mainly at the level of muscle fibers, in particular on the sarcolemma and within some myofibers. Of note, in *LMNA*-RM group, we observed a TLR9 positive immunoreactivity higher in dystrophic than in myopathic muscle biopsies (Figure 3 and Supplementary Figure 2). A TLR9 positive staining was observed also in the endomysial space, on capillaries and small blood vessels and to a lesser extent at the level of inflammatory cells, especially in sIBM (Figure 3).

In *LMNA*-RM muscles, TLR4 was strongly expressed on the sarcolemma of some myofibers and on capillaries and small blood vessels (Figure 4). Sporadic muscle fibers showed a positive staining for the receptor at the level of the sarcoplasm, whereas few inflammatory cells were TLR4 positive. sIBM muscles showed a strong immunopositivity for TLR4; the receptor was expressed on the sarcoplasm and within some muscle fibers, in particular in small sized degenerating/regenerating myofibers (Figure 4), and in many inflammatory infiltrates. In FSHD a TLR4 positive staining was mainly observed on the sarcolemma of some myofibers and on capillaries. Rare muscle fibers expressed TLR4 also in the sarcoplasm.

In non-myopathic controls the staining for the all three TLRs was absent or rarely observed at the level of small blood vessels (Figures 2–4).

### Macrophage identification among inflammatory cells in *LMNA*-RM muscle tissue

Macrophages represent the most frequent inflammatory cells in chronic muscle damage. Widespread necrosis of muscle cells support the persistence of macrophage subtypes, mainly of M2, that contribute to excessive connective tissue deposition and muscle fibrosis [22].

**Figure 5.**
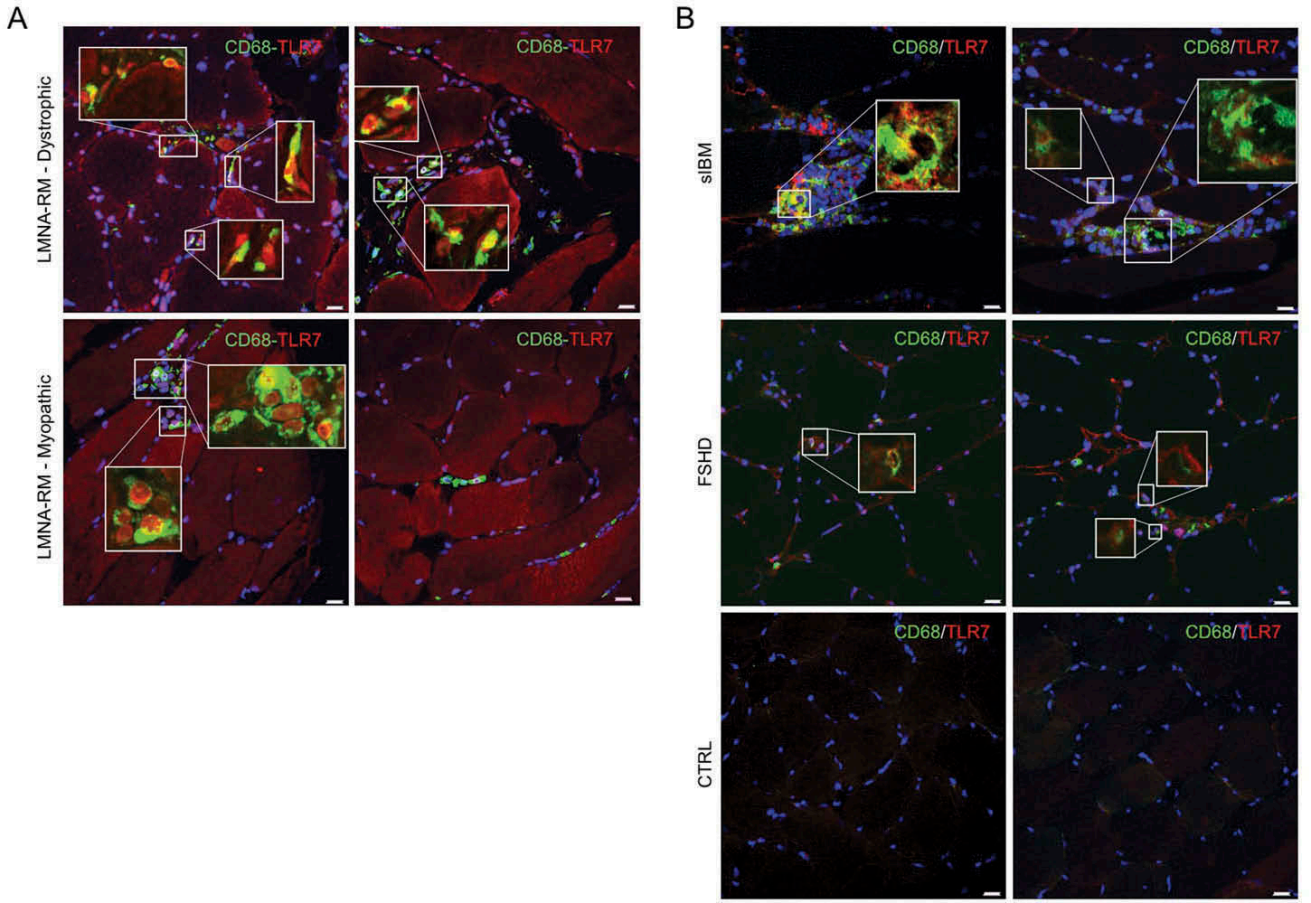
Quantification of macrophages residing in *LMNA*-RM muscle tissue. (a) Immunofluorescence showed the presence of CD68 (green) positive macrophages among discernable muscle inflammatory cells scattered or localized close to connective tissue deposits in *LMNA*-RM (mean ± SD: 11.83% ± 8.06). Colocalization between CD68 and TLR7 was highlighted in the insets. (b) Among pathological muscle samples, sIBM showed the highest percentage of CD68-TLR7 double positive cells (mean ± SD: 23.73% ± 6.95) whereas the lowest values of colocalization between the two molecules was observed in FSHD muscles (mean ± SD: 8.92% ± 1.72). Original magnification, X40 and X120 in the insets; bars: 20 μm.

In view of these considerations and since macrophages represent the main source of TLR7 besides dendritic cells [23], we evaluated the presence of macrophages in *LMNA*-RM muscle tissue. Immunofluorescence showed a relatively high percentage of CD68-TLR7 double positive cells than expected among inflammatory cells in *LMNA*-RM muscle (mean ± SD: 11.83% ± 8.06%) (Figure 5A). CD68 positive macrophages were scattered in the tissue or localized close to degenerated muscle fibers and connective tissue deposits, mainly in the dystrophic form of *LMNA*-RM. In sIBM, where the presence of inflammatory cells is usually abundant [24,25], the percentage of CD68-TLR7 double positive macrophages was 23.73% (± 6.95%), whereas in FSHD muscle tissue it was 8.92% (± 1.72%) (Figure 5(b)). No positive staining was observed for the macrophage marker CD68 in non-myopathic control samples (Figure 5(b)).

Additionally, further characterization of inflammatory cells in *LMNA*-RM muscles showed the presence of a few percentage of T lymphocytes (mainly CD4 positive T cells), some CD11c positive myeloid  dendritic cells and rare CD138 positive plasma cells (Supplementary Figure 3).

### TGF-β expression in *LMNA*-RM muscle tissue

**Figure 6.**
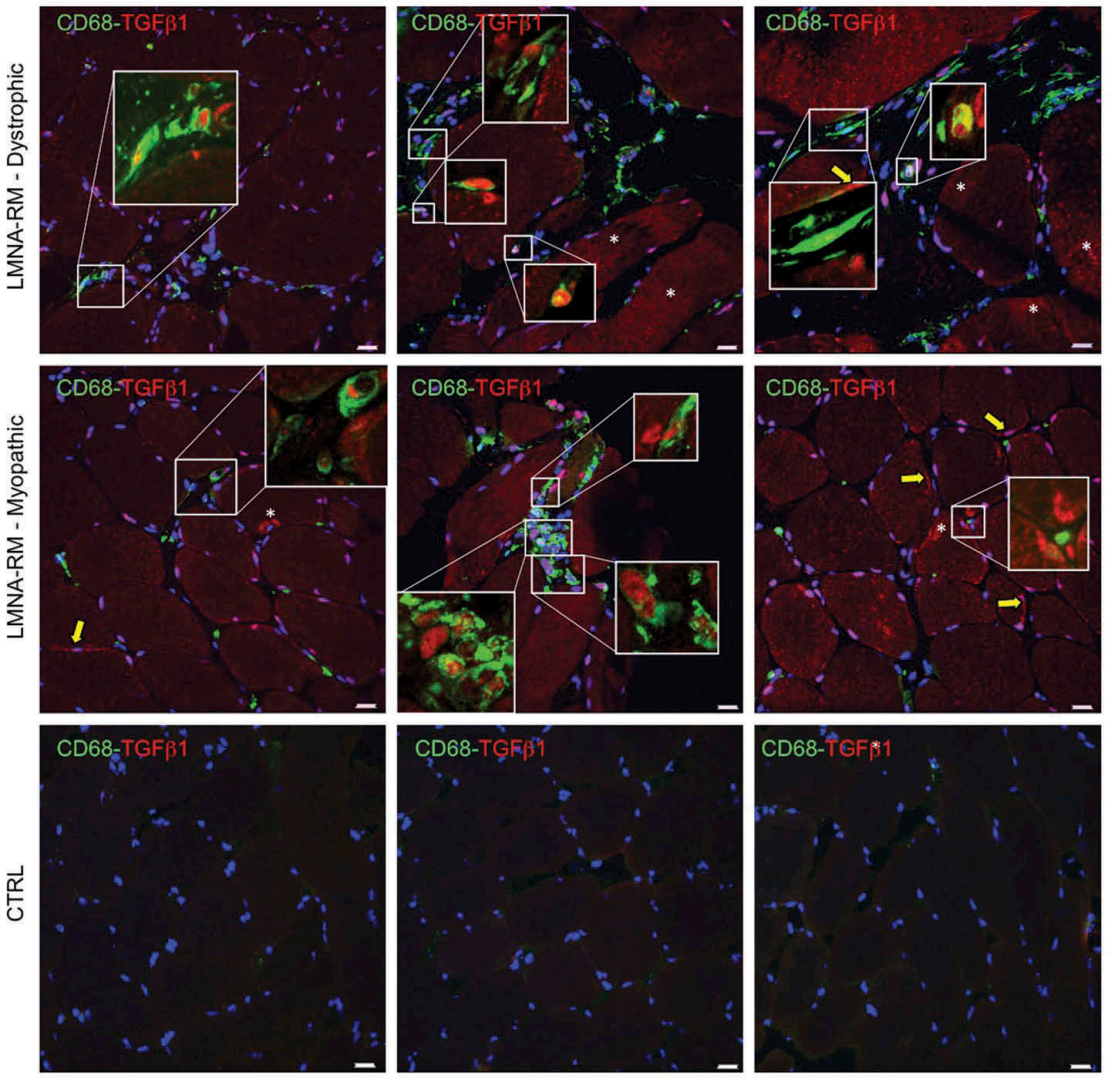
Expression and localization of TGF-β1 in *LMNA*-RM muscle tissue. Double immunofluorescence showed a TGF-β1 (red) positive staining subjacent to sarcolemma (yellow arrows) or in the sarcoplasm (white asterisks) of some muscle fibers (mean ± SD: 31.64% ± 11.50). A TGF-β1 positive immunoreactivity was observed in the majority of CD68 (green) positive macrophages (mean ± SD: 67.60% ± 5.62), as shown in the insets. Absence of CD68 and/or TGF-β1 staining was observed in non-myopathic muscles (CTRL). Original magnification, X40 and X120 in the insets; bars 20 μm.

By confocal microscopy, we evaluated the expression and localization of TGF-β1 in *LMNA*-RM muscle biopsies. As previously reported for DMD [26,27], we observed that TGF-β1 was mainly expressed in the sarcoplasm or subjacent to sarcolemma of some non-necrotic muscle fibers (Figure 6): in particular, in dystrophic *LMNA*-RM muscles TGF-β1 expression seems to be homogeneously distributed in the sarcoplasm (Figure 6, upper panel); in myopathic *LMNA*-RM muscles it seems to be more located in the periphery of muscle fibers in close proximity to CD68 positive macrophages (Figure 6, middle panel). Among the macrophage population, the percentage of TGF-β1-expressing CD68 positive macrophages was 67.60% (± 5.62). No positive staining for TGF-β1 was observed in non-myopathic control samples (Figure 6).

## Discussion

Laminopathies represent a heterogeneous group of diseases with overlapping phenotypes that are caused by mutations in the nuclear envelope proteins lamins A and C. The mechanisms by which the nearly ubiquitously expressed lamins A and C (and also emerin) can cause such tissue-specific phenotypes are not well understood, although different hypotheses have been suggested [2,28].

Muscle inflammatory findings have been already reported in *LMNA*-RM, especially in patients with early presentation [4–7]. As supposed for DMD [8], the genetic defect might determine also in *LMNA*-RM the activation of an innate immune response, leading to inflammation and contributing to myofiber necrosis and fibrosis. Recent reports showed that mutations in lamins and lamin-binding proteins are usually associated with the activation of the transcription factor NF-κB and secretion of high levels of pro-inflammatory cytokines [29,30]. Considering that NF-κB activation represents an important step in TLR signaling pathways [31], and taking into account the involvement of TLR7 and TLR9 in the pathogenesis of dystrophin-deficient skeletal muscle [8], in this study we proposed to evaluate the presence and the expression levels of endosomal TLRs in *LMNA*-RM muscle tissue. We found a significant increase in the mRNA expression levels of TLR7 and TLR9 in *LMNA*-RM muscles, when compared to controls and to other forms of myopathies such as FSHD. *LMNA*-RM muscle biopsies showed intense TLR7 and TLR9 staining, mainly localized at the level of muscle fibers and of discernible mononuclear inflammatory cells. Within *LMNA*-RM muscle group, we did not observe a significant difference in the TLR7/9 expression between dystrophic and myopathic histological phenotype. In *LMNA*-RM dystrophic muscles, we found a slight increase in TLR7/9 immunoreactivity compared to the myopathic subgroup; this was probably due to the presence of necrotic fibers and/or connective tissue deposits, less common in the myopathic muscles. Likewise, neither the different age of onset of the disease nor the different time lapse between the onset and the biopsy, seem to affect TLR7/9 expression.

Altogether our findings suggest that, independently from the clinical and histological phenotype, alteration of the nuclear lamin architecture might have a more substantial role in the activation of a TLR7/9-dependent immune response compared to other forms of neuromuscular genetic disorders, such as FSHD.

In most forms of skeletal muscle injury the main inflammatory cell type, expressing TLR7/TLR9, is represented by macrophages [13,32]. They release several cytokines, chemokines and extracellular proteases that affect satellite cell behavior, extracellular matrix remodeling and various aspects of skeletal muscle repair [32]. In order to better understand if macrophages were the main source of TLR7/TLR9 in the *LMNA*-RM muscle tissue, we characterized the few discernable immune cells in our group of *LMNA*-RM muscle biopsies. Indeed, besides few CD11c positive myeloid dendritic cells and CD4 positive T lymphocytes and rare CD138 positive plasma cells, the majority of mononuclear immune cells was composed by CD68 positive macrophages.

A recent study, performed in a *LMNA* mouse model, demonstrated that a mutation in the *LMNA* gene cause structural lesions of the nuclear laminae in macrophages [33]. These nuclear changes induced a modification of the adhesive properties of macrophages and promote the production of TGF-β and TNF-α. Furthermore, considering that the persistent activation of TLR7/9 has been shown to push macrophages into phagocytic long-lived cells, characterized by a decrease capacity of antigen presentation and a pronounced inclination to produce TGF-β [23], we proposed to evaluate the expression of TGF-β also in *LMNA*-RM muscle biopsies. We found that approximately 30% of muscle fibers showed a TGF-β positive immunoreactivity, as well as the majority of CD68 positive macrophages. Therefore, these results suggest a contribution of TGF-β1 to muscle damage not only in DMD, but also in *LMNA*-RM muscle tissue.

In summary, our data add a further insight in the pathogenesis of *LMNA*-RM, demonstrating that *LMNA*-RM muscle tissue is characterized by the over-expression of TLR7 and TLR9, probably due to the release of danger signals, as supposed for DMD, or to a NF-κB signaling impairment. Our findings suggest that TLR persistent activation, in addition to mutations in *LMNA* gene, induces substantial modifications in the behavior not only of muscle cells but also of macrophages residing in *LMNA*-RM muscle tissue. Both cell types are pushed to secrete several pro- and anti-inflammatory cytokines, mainly TGF-β, promoting an impairment in muscle regeneration and favoring muscle fibrosis. Therefore, blocking or modulation of TLR7/9 signaling pathway may have important therapeutic implications for *LMNA*-RM.

## Patients and methods

### Patients and muscle biopsies

Nine patients affected by *LMNA*-RM clinically and genetically defined (mean age at muscle biopsy: 30.37 years, ranging from 1.5 to 54), 8 patients with clinically-defined or probable sporadic inclusion body myositis (sIBM) (mean age at muscle biopsy: 66.12 years, ranging from 57 to 79), according to the European Neuromuscular Centre diagnostic criteria [24], 6 patients affected by facio-scapulo-humeral muscular dystrophy (FSHD) (mean age at muscle biopsy: 45.71 years, ranging from 34 to 66) and 14 controls (mean age at muscle biopsy: 50.82 years, ranging from 31 to 63), who underwent biopsy for diagnostic purposes, but without myopathy confirmation, were included in the study. Clinical and molecular features of *LMNA*-RM patients are shown in Table 1. Muscle tissues were obtained by open or needle biopsy from quadriceps or biceps brachii, immediately frozen in precooled isopentane and then stored in liquid nitrogen. This study was performed according to the institutional review board-approved clinical protocols. Each individual provided written informed consent to muscle biopsy for diagnosis and research purposes.

**Table 1.** *LMNA*-RM patients’ clinical and genetic data.

Patient	Disease	Age at onset (y)	Age at muscle biopsy (y)	Phenotype	Mutation
#1	LMNA-LGMD1B	23	54	Myopathic	c.1609-108G>A
#2	LMNA-CMD	Birth	1	Myopathic	R249W
#3	LMNA-LGMD1B	5.5	34	Dystrophic	R25G
#4	LMNA-LGMD1B	32	35	Dystrophic	R377H
#5	LMNA-LGMD1B	33	46	Myopathic	R377H
#6	LMNA-CMD	Birth	26	Dystrophic	R527C
#7	LMNA-EDMD2	37	42	Myopathic	R644C
#8	LMNA-CMD	Birth	5	Myopathic	L35del
#9	LMNA-CMD	Birth	1.5	Myopathic	E358K

### RNA extraction and cDNA synthesis

Total RNA was extracted from 10 to 20 mg of frozen muscle tissue from 9 *LMNA*-RM patients, 8 patients affected by sIBM, 6 patients with FSHD and 14 controls using TRIzol® Reagent (Invitrogen, Thermo Fisher Scientific, *#*15596018), followed by DNA-free™ DNA Removal kit (Ambion, Thermo Fisher Scientific, *#*AM1906). For mRNA amplification, random-primed cDNA was prepared using the SuperScript® VILO cDNA Synthesis kit (Invitrogen, Thermo Fisher Scientific, #11754–050) following the manufacturer’s instructions and stored at −20°C pending polymerase chain reaction (PCR) amplification.

### Quantitative real-time PCR (qRT-PCR)

cDNA amplification and data acquisition were performed with a ViiA™ 7 Real-Time PCR System (Applied Biosystems, Thermo Fisher Scientific), using pre-designed functionally-tested assays: TLR7: Hs00152971_m1; TLR9: Hs00152973_m1; TLR4: Hs00152939_m1 (Applied Biosystems, Thermo Fisher Scientific, #4331182). All the reactions were done in triplicate and normalized to the reference gene GAPDH (Hs99999905_m1) (Applied Biosystems, Thermo Fisher Scientific, #4331182).

### Immunofluorescence and confocal microscopy

Six µm-thick cryostat cut muscle sections from 4 *LMNA*-RM, 4 sIBM, 3 FSHD and 3 controls, fixed for 10 minutes with 4% paraformaldehyde and permeabilized for 10 minutes with 0.1% Triton X-100, were incubated with 5% bovine serum albumin/phosphate buffered saline (BSA/PBS) for 30 minutes at room temperature to avoid nonspecific binding. The slides were then incubated overnight at 4°C with various combinations of two primary antibodies diluted in 1% BSA/PBS at the following dilutions: 1:50 for anti-TLR7 (Enzo Life Sciences, #ALX-210–874-C100), 1:20 for TLR4 and TLR9 (both from Santa Cruz Biotechnology, # sc-10741 and sc-25468), 1:250 for anti-CD68, 1:10 for anti-CD4, 1:10 for anti-CD3, 1:50 for anti-CD138 (all from Dako, #M0876, clone PG-M1; #M0716, clone MT310; #M0835, clone UCHT1; #M7228, clone MI15), 1:20 for anti CD11c (BD Pharmingen™, #550375) and 1:200 for anti TGF-β1 (Abcam, #ab53169).

After three washes with PBS, the slides were incubated with two secondary antibodies, 1:400 Alexa fluor 488-conjugated goat anti-mouse IgG [H + L], and 1:600 Cy™ 3 AffiniPure Goat Anti-Rabbit IgG [H + L] (both from Jackson Immunoresearch Laboratories, #115–545-003 and #111–165-003), for 1 hour at room temperature. As negative control, sections were incubated with isotype-specific nonimmune IgG (Dako, #), rabbit serum (from our laboratory, diluted as were primary antibodies), and DAPI (4ʹ,6-Diamidino-2-Phenylindole, Dihydrochloride, #D1306, 300nM, Thermo Fisher Scientific) as nuclear marker.

The slides were mounted with FluorSave™ Reagent (Merck Calbiochem, #345789), sealed and dried for 1 hour at room temperature. Images were captured with a Nikon Eclipse TE2000-E confocal laser-scanning microscope and analyzed with EZ-C1 3.70 imaging software (Nikon). Positive fluorescence staining was quantified by image analysis performed using ImageJ software (http://rsb.info.nih.gov/ij). Single- or double-positive cells were counted on 5 adjacent fields per section at x40 magnification, and expressed as ratio between the number of positive cells and that of DAPI positive nuclei x 100; the results were reported as mean percentage and standard deviation (SD).

### Statistical analysis

The Kruskal-Wallis non-parametric test, with Dunn’s nonparametric comparison post hoc test, was used to assess differences in mRNA levels in disease groups and controls, since they were not normally distributed. Unpaired *t*-test was used when two groups were compared. Differences were considered significant at *p *< 0.05. The analyses were performed with GraphPad Prism version 5.0 (GraphPad Software, San Diego, CA) for Mac.
