# Supplementary material for: Up-regulation of Toll-like receptors 7 and 9 and its potential implications in the pathogenic mechanisms of LMNA-related myopathies
Source: Nucleus. 2018 Oct 3;9(1):398–409. doi: 10.1080/19491034.2018.1471947 (PMC7000140; doi:10.1080/19491034.2018.1471947)
Supplement: Supplemental Material [file kncl-09-01-1471947-s001.docx]

**MS KNCL-2017-0031**

**Supplementary Figures**

*Supplementary Figure 1.* Comparison between dystrophic and myopathic *LMNA*-RM muscle tissues for TLR7 expression.

*Supplementary Figure 2.* Comparison between dystrophic and myopathic *LMNA*-RM muscle tissues for TLR9 expression.

*Supplementary Figure 3.* Characterization of inflammatory cells in *LMNA*-RM muscles.


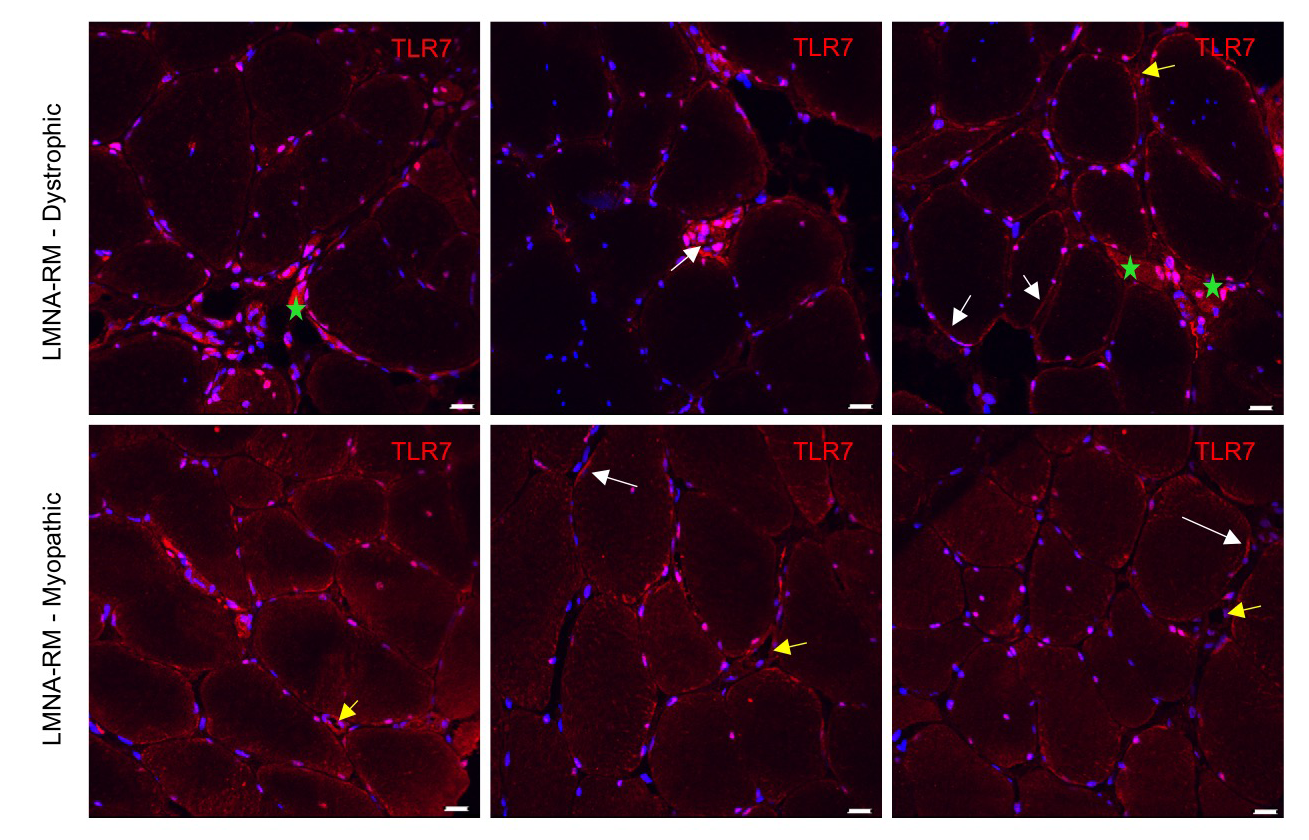


**Supplementary Figure 1.** Comparison between dystrophic and myopathic *LMNA*-RM muscle tissues for TLR7 expression. TLR7 was highly expressed in *LMNA*-RM muscle biopsies, especially in dystrophic patients. TLR7 was detected at the level of blood vessels and capillaries (*yellow arrows*), on the sarcolemma of some muscle fibers (*white arrows*) and within some myofibers (*green stars*). Regenerating/degenerating muscle fibers in dystrophic patients were particularly positive for TLR7 (*green stars*) (upper panel). Nuclei are stained with DAPI (blue). Original magnification, X40. Bars: 20 μm.

**
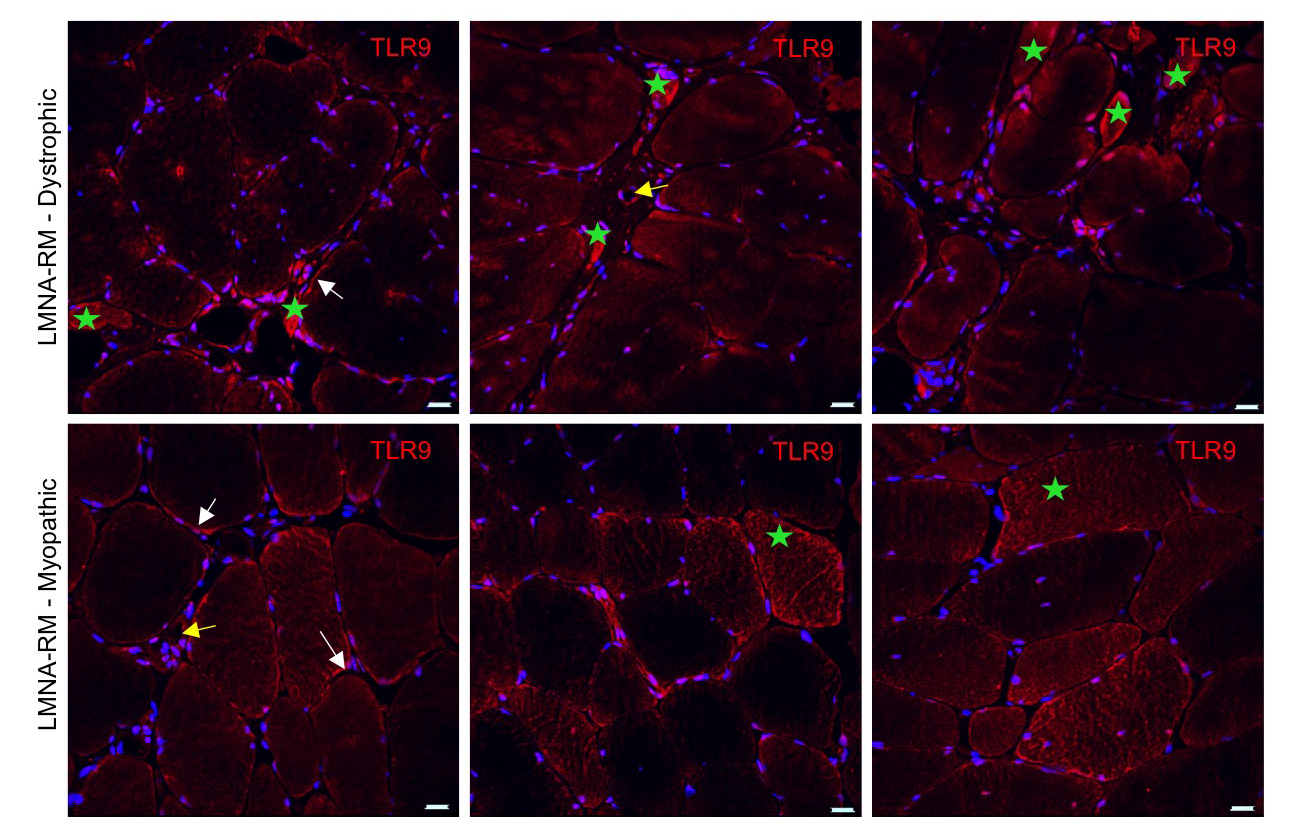
**

**Supplementary Figure 2.** Comparison between dystrophic and myopathic *LMNA*-RM muscle tissues for TLR9 expression. TLR9 was highly expressed in *LMNA*-RM muscle biopsies, especially in dystrophic patients. TLR9 was detected at the level of blood vessels and capillaries (*yellow arrows*), on the sarcolemma of some muscle fibers (*white arrows*) and within some myofibers (*green stars*). Regenerating/degenerating muscle fibers in dystrophic patients were particularly positive for TLR9 (*green stars*) (upper panel); in the surrounding tissue of dystrophic *LMNA*-RM, myofibers showed an immunoreactivity for the receptor mainly distributed at the periphery of muscle cell (upper panel), whereas in myopathic muscles TLR9 was more homogeneously distributed in the sarcoplasm (lower panel). Nuclei are stained with DAPI (blue). Original magnification, X40. Bars: 20 μm.

**
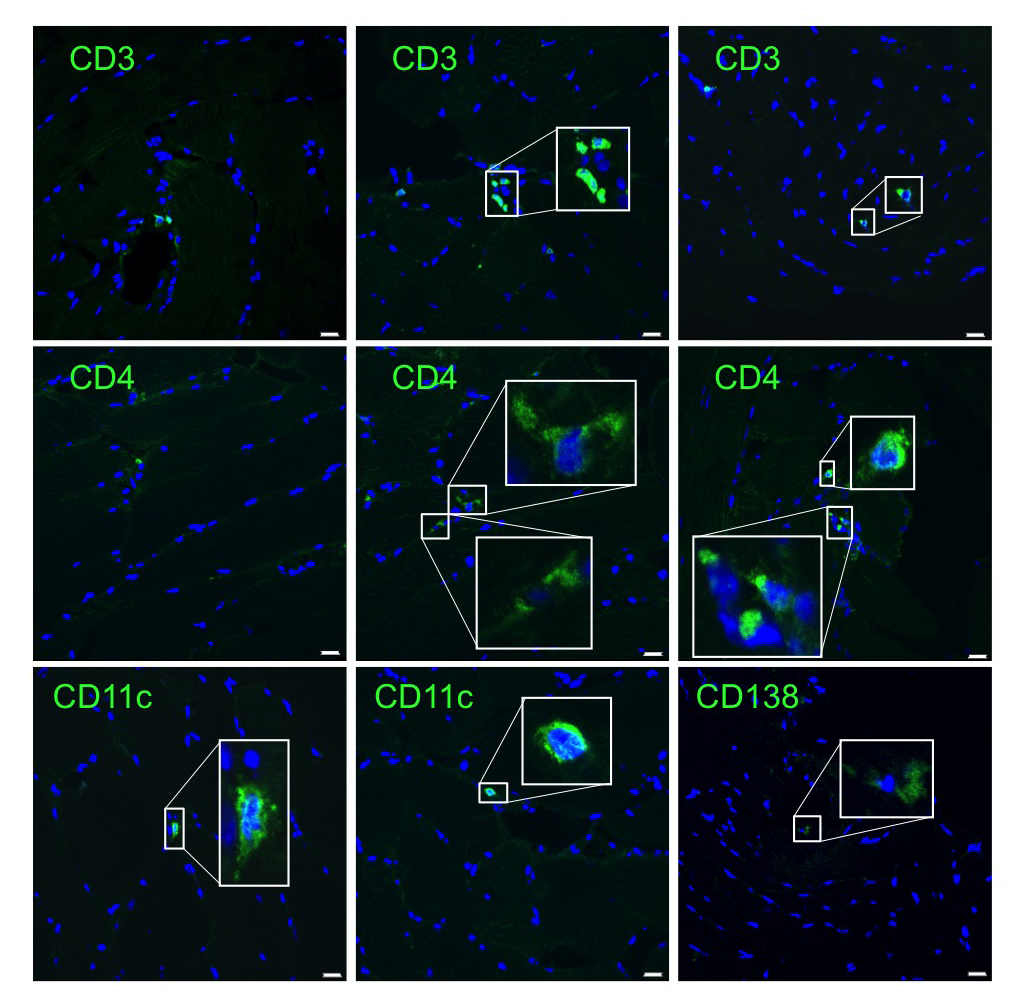
**

**Supplementary Figure 3.** Characterization of inflammatory cells in *LMNA*-RM muscles. Few CD3 positive T lymphocytes, mainly of CD4 phenotype, some CD11c positive dendritic cells and very rare CD138 positive plasma cells were detected in *LMNA*-RM muscle biopsies. Nuclei are stained with DAPI (blue). Original magnification, X40. Bars: 20 μm.
